# Assessment of Metacognition and Reversal Learning in Parkinson’s Disease: Preliminary Results

**DOI:** 10.3389/fnhum.2018.00343

**Published:** 2018-09-11

**Authors:** Carlos Trenado, Matthias Boschheidgen, Julia Rübenach, Karim N’Diaye, Alfons Schnitzler, Luc Mallet, Lars Wojtecki

**Affiliations:** ^1^Institute of Clinical Neuroscience and Medical Psychology, Medical Faculty, Heinrich Heine University Düsseldorf, Düsseldorf, Germany; ^2^Center for Movement Disorders and Neuromodulation, Department of Neurology, Medical Faculty, Heinrich Heine University Düsseldorf, Düsseldorf, Germany; ^3^Department of Psychology and Neurosciences, Translational Neuromodulation Unit, Leibniz Research Centre for Working Environment and Human Factors, TU Dortmund, Dortmund, Germany; ^4^Institut du Cerveau et de la Moelle Épinière, Hôpital Pitié Salpêtrière, Paris, France

**Keywords:** behavioral adaptation, reversal learning, subthalamic nucleus, levodopa, Parkinson’s disease, metacognition

## Abstract

Reversal learning (RL) has been widely used for assessment of behavioral adaptation, impulsivity, obsession, and compulsion in healthy controls as well as people suffering from psychiatric and neurological disorders such as Parkinson’s disease (PD). Nevertheless, studies addressing high cognitive functions such as metacognition in PD are scarce. Here, we address for the first time the effect of levodopa and PD on metacognition within the framework of a RL paradigm. In agreement with previous reports, PD patients exhibited reversal shifting impairment with respect to healthy controls (CTRL) regardless of medication condition (MED-ON and MED-OFF), which was supported by a well-known model of learning conditioning (Rescorla–Wagner). In spite that we found a significant association between accuracy and decision confidence level for MED-OFF and CTRL, analysis of metacognitive sensitivity assessed by type 2 signal detection theory (SDT) revealed only a significant underperformance for patients without medication (MED-OFF). This finding points toward a non-compromising positive effect of dopaminergic medication on metacognition for PD.

## Introduction

Dopaminergic medication and deep brain stimulation (DBS) are established treatments for Parkinson’s disease (PD), which not only provide sustained motor effects (Groiss et al., 2009; Vijverman and Fox, 2014) but also have been implicated in modulating cortico-striatal circuitry with an indirect effect in cognitive and behavioral abilities (Calabresi et al., 2015; Da Cunha et al., 2015). In relation to this, the way both treatments operate in impulsive behavior as reflected by behavioral adaptation and metacognition in PD patients remains poorly understood.

In order to address behavioral adaptation in PD patients, previous studies considered adaptation toward reward contingency changes by using a probabilistic reversal learning (RL) task. Such task requires subjects to discriminate between two stimuli on the basis of feedback with a specified probabilistic error and continuous monitoring of contingency changes (reversals). In particular, it has been reported that l-dopa impairs performance and facilitates task-switching (Swainson et al., 2000; Cools et al., 2001), while other studies emphasized enhancement of reward seeking behavior and RL impairment (Graef et al., 2010). Moreover, it has been reported that dopaminergic medication impairs reversal shifting depending on the motivational valence of unexpected outcomes (Cools et al., 2006), and performance on the extinction phase of a RL/extinction task with a corresponding improvement under effect of deep brain stimulation of the subthalamic nucleus (DBS-STN) (Funkiewiez et al., 2006). For PD patients without medication, distinct pre- and post-reversal deficiencies dependent of the difficulty of the task were revealed (Peterson et al., 2009).

With regard to neural mechanism of RL, abnormal changes in the ventral frontostriatal circuitry have been implicated with deficits in reversal shifting (Cools et al., 2002). In PD patients, it has also been emphasized the role of the nucleus accumbens in dopaminergic modulation resulting from performance of a RL task (Cools et al., 2007).

Focusing on metacognition, which reflects higher order processing of cognitive processes engaged in learning and self-awareness, it has been reported that a form of metamemory, the tip-of the thong phenomenon, is not compromised in PD patients (Oh-Lee et al., 2012). In addition, it has been reported that self-awareness of impulse-control disorder (ICD) is comparable or increased in PD patients with ICD compared to those without ICD (Mack et al., 2013). Furthermore, olfactory metacognition has been shown to be impaired in PD patients, as reflected by less accurate assessment of patient’s own ability in identifying olfactory stimuli (White et al., 2016). Notably, maladaptive metacognitive style has been implicated with high levels of distress in PD (Allott et al., 2005).

Building on previous findings, we addressed metacognition within a RL paradigm in PD patients by considering two conditions: with or without dopaminergic medication (MED-ON and MED-OFF). We expected RL impairment and differential metacognitive judgment between PD patients and healthy controls (CTRL).

## Materials and Methods

### Patients and Healthy Controls

Ten PD patients (4 female and 6 male) and 10 age-matched healthy controls (5 female and 5 male) with no previous experience in performing a RL task participated in this study. Dementia and major depression were ruled out on the basis of psychological assessment Mattis Dementia Rating Scale (MDRS) with a cut-off score of ≤132 (Mattis, 1988) and the Beck Depression Inventory (BDI) with a cut-off score of ≥18 (Beck, 1987). Furthermore, patients suffering from diseases that may have interfered with their performance were excluded. **Table 1** gives an overview of psychological scores and age for patient and healthy controls. Levodopa equivalent dose was calculated as described elsewhere (Tomlinson et al., 2010). The study was in compliance with the Helsinki Declaration and was approved by the ethics committee of the University Hospital Düsseldorf (Study No. 3209). Patients and healthy subjects that participated in the study signed an informed consent form.

**Table 1:** Patient characteristics: age (years), disease duration with respect to the date of operation (years), mean score of the Beck depression inventory (BDI), mean score of the positive and negative affect schedule (PANAS), mean score of the Mattis dementia rating scale (MDRS), UPDRS OFF: Score of the motor part of the UPDRS without medication.

	Age	Disease duration	BDI	PANAS (±)	MDRS	UPDRS OFF/ON	LED
Patient (*n* = 10)	62.8 ± 7.4	7.8 ± 4.73	7.6 ± 4.8	27.9 ± 5.3/15.7 ± 6.0	139.3 ± 3.3	26.4 ± 8.9/17.1 ± 8.7	1032 ± 350.6
Controls (*n* = 10)	64.3 ± 7.4	Na	5.8 ± 3.1	36.7 ± 5.3/13.8 ± 3.9	141.7 ± 1.3	Na	Na


*UPDRS ON: Score of the motor part of the UPDRS with medication, Levodopa equivalent dose (LED). UPDRS OFF: Score of the motor part of the UPDRS without medication.*

### Study Design

At arrival, subjects signed the informed consent form. Next, neuropsychological screening was conducted to determine the suitability of participants, i.e., absence of dementia and depression symptoms. Subsequently, participants sat comfortably and were instructed on how to perform the RL task. For PD patients, two conditions were tested in randomized order: MED-OFF (patients performed the RL task without dopaminergic medication) and MED-ON (patients performed the RL task under effect of dopaminergic medication). In particular, MED-ON was triggered by a dose of l-dopa (7/10 patients) or by dopamine agonists in combination with l-dopa (3/10 patients) (**Table 2**). Note that 3/10 patients were tested with 1 day difference between conditions, while 7/10 patients were tested the same day with a between condition time interval of at least 3 h to avoid fatigue. For patients (3/10) that were first tested in MED-ON, MED-OFF testing took place the next day (at least 12 h passed since the last medication) due that MDS-UPDRS in our clinic is routinely performed in patients that withdrew from dopaminergic medication the night before. For patients (7/10) that were first tested in MED-OFF, MED-ON state was triggered by a dose of levodopa LT administered 1 h before behavioral testing.

**Table 2:** Full medications for each patient including dopaminergic medications used for the condition MED-ON.

Patient	(On)/full medications
1	(Levodopa 100 mg/Carbidopa 25 mg//Entacapone 200 mg)/Piribedil 50 mg
2	(Madopar LT 200 mg)/L-dopa 150 mg/Benserazid 32.5 mg /Rasagilin 1 mg/Ropinirole 8 mg
3	(Madopar LT 200 mg)/L-dopa 100 mg/Carbidopa 25 mg/Entacapone 200 mg/Piribidel 50 mg/Amantadine 150 mg/Rasagilin 1 mg
4	(Madopar LT 150 mg/Benzerazid 12.5 mg)/Safinamide 50 mg/ Madopar 12.5 mg/ Ropinirole 8 mg/Madopar dep 100 mg
5	(Madopar LT 200 mg)/Levodopa 200 mg/Carbidopa 25 mg/Entacapone 200 mg/Levodopa 100 mg/Benserazid 25 mg/Levodopa 50 mg/Benserazid 12.5 mg/Rapimisol 0.5 mg
6	(Madopar LT 200 mg)/L-dopa 100 mg/Benserazid 25 mg/Opicapone 50 mg/Duodopa pump 3.9 ml/h
7	(Levodopa 150 mg/Carbidopa 37.5 mg/Entacapone 200 mg)/L-dopa 100 mg/Benserazid 25 mg/Pramipexol 0.7 mg/Rivastigmine 1.5 mg
8	(Madopar LT 200 mg)/L-dopa 100 mg/Benserazid 25 mg
9	(Madopar LT 200 mg)/L-dopa 200 mg/Benserazid 50 mg/Rotigotine 2 mg (24 h)
10	(Madopar LT 300 mg)/L-dopa 150 mg/Carbidopa 37.5 mg/Entacapone 200 mg


### Neuropsychological Tests

Depression was assessed with the BDI (score range: 0–63). In accordance to our previous PD studies (Keitel et al., 2013), a cut-off score of ≥18 was selected for clinically relevant depression before study participation.

We assessed positive (PA) and negative affect (NA) with the PANAS questionnaire (Watson et al., 1988) (score ranges for PA and NA: 10–50). PA reflects the extent to which individual experiences a positive disposition, while NA reflects subjective distress and unpleasant engagement.

Dementia was assessed with the MDRS (maximum score: 144). A cut-off above 132/144 for absence of dementia was adopted in accordance to previous reports (Matteau et al., 2012).

### Motor Function Tests

MDS-UPDRS scores were obtained after each testing condition. All patients in our study showed no freezing symptoms, however, (3/10) patients (**Table 2**, patients: 2, 6, and 10) displayed dyskinesia.

### Reversal Leaning Task

The RL task utilized in the present study was implemented by using the Psychotoolbox software^1^. Stimuli consisted of pairs of Hiragana characters that were presented on a 19″ computer monitor placed 50 cm apart from the participant. The RL task included two types of trials, e.g., trials with and without metacognitive assessment, which we termed as metacognitive and standard trials, respectively. The order of trial presentation was randomized.

**FIGURE 1:**
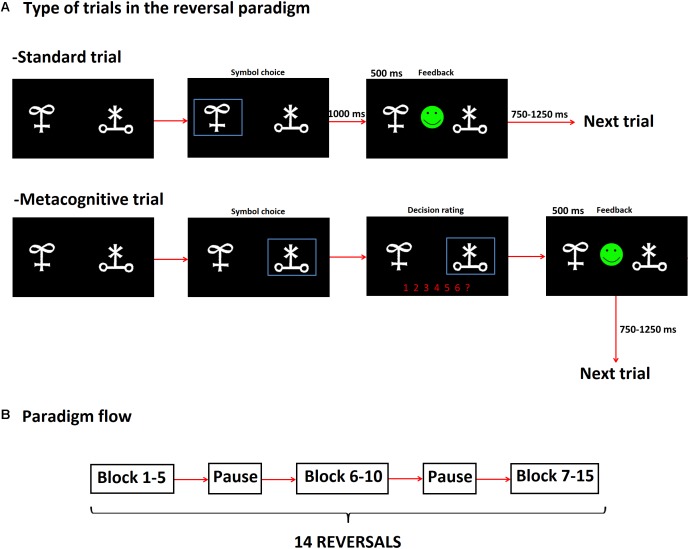
Scheme of the reversal learning (RL) task that incorporated metacognitive assessment. **(A)** The RL task included trials with and without metacognitive assessment, which we termed as Metacognitive and Standard trials, respectively. The order of presentation of such trials was random, **(B)** Paradigm blocks: each subject completed 15 blocks (divided in three sections), after block 5 and 10 there was a pause. The three blocks comprised 14 reversals in total. The criterion to reach reversal was as follows: a subject needed to complete 10 consecutive right trials. After such criterion was fulfilled, there was a 25% chance for reversal to occur in the next five trials as long as the subject kept on performing consecutive right trials. If subjects chose the wrong symbol before reaching the specified number of consecutive right trials, they had to begin again.

For standard trials, subjects selected one of two symbols displayed in the screen by pressing a key on a keyboard. Stimuli remained on the screen till subjects responded. Feedback (happy or sad smiley) was presented for 500 ms after participant’s response. A new stimulus was presented at a random time between 750 and 1250 ms (**Figure 1A**). For metacognitive trails, subjects were additionally asked to provide a decision’s confidence rating on a scale from 1 to 6 (1: less confident and 6: very confident) before feedback was provided (**Figure 1A**).

Contingencies between two symbols were fixed to a rate 80/20, namely the rewarded symbol was followed by positive feedback except in 20% of the trials. Probabilistic error was defined for trials when subjects chose the rewarded symbol and subsequently received negative feedback. A reversal criterion was set up by 10 consecutive right trials. As we used a probabilistic RL task, a right trial was not always followed by positive feedback in each trial. The criterion for reversal was as follows: after completion of 10 right trials, a reversal was set up to occur with probability 25% in the next five trials as long as the subject kept on performing consecutive right trials. If subjects chose the wrong symbol before reaching the specified number of consecutive right trials, they had to begin again. Subjects were instructed to change their decision only when they were absolutely sure that contingencies had changed, taking into consideration that probabilistic error could be present.

Each subject completed 15 blocks that consisted of 14 reversals in total (the average number of standard trials performed by CTRL, MED-ON, and MED-OFF subjects 361.40 ± 61.91, 518.40 ± 93.52, and 575.60 ± 241.64, respectively, the average number of metacognitive trials for CTRL, MED-ON, and MED-OFF were 53.20 ± 9.43, 75.40 ± 14.89, and 86.5 ± 38.09, respectively) (**Figure 1B**). There was a break after block 5 and 10. After each break, the two symbols used for stimuli were replaced by new ones. Probabilistic error could not occur in the first three trials after reversal. Three or less consecutive probabilistic errors were possible during blocks so that subjects could not follow a specific strategy (e.g., change after second negative feedback). The experiment time varied between 30 and 60 min depending on subject’s performance.

### Learning Model

To address learning rate differences between PD patients and healthy controls in relation to decision making as reflected in our behavioral data, we used the Rescorla–Wagner learning model (RWLM) (Rescorla and Wagner, 1972). In particular, such prediction-error based model captures learning [indexed by the learning rate parameter (α)], that occurs if the choice made does not match expectation indexed by the prediction error (λ_*t*−1_ − *V*_*a,t*−1_), as given by the formula:

$$V_{a,t}\;=\;V_{a,t-1}+\alpha (\lambda_{t-1}-V_{a,t-1})$$

where V _a,t_ is the value of stimulus “a” at trial *t* and λ_*t*−1_ is the outcome at trial t - 1. A soft-max function was utilized to model how learning parameters are translated into a choice as reflected by the formula:

$$p(a)\;=\;\frac{e^{\left(\beta *Va\right)}}{\sum_{i}e^{\left(\beta *Vi\right)}}$$

where *p(a)* denotes the probability of selecting stimulus “a” and β denotes the decision parameter. Note that a large value of β indicates that choices becomes more “deterministic,” i.e., subjects are more likely to pick the option with the highest reward, while a small value of β indicates that choices become more exploratory, i.e., subjects are more likely to switch between options. The MATLAB code used for the RWLM model is a modified version of the code provided by Hanneke den Ouden^2^.

### Data Analysis

#### Behavioral Data

For analysis of the behavioral data, we considered the following RL parameters: (1) mean number of trials to reach reversal; (2) number of consecutive errors after reversal; (3) random switches, which refers to trials in which subjects randomly switched their choice in spite of getting positive feedback; (4) strategy change after probabilistic error (PE), which quantifies trials with change in decision occurring after PE. Focusing on performance, the following parameters were considered: (5) response time (RT), and (6) accuracy. For metacognition, we included: (7) mean confidence level.

#### Metacognitive Data

We addressed the relationship between confidence rating scale and accuracy by statistical tests (Chi-square test). Metacognition type 2 sensitivity (meta-d), i.e., the efficacy with which observers’ confidence ratings discriminated between their own correct and incorrect stimulus classification, was assessed by type 2 signal detection theory (SDT) (Maniscalco and Lau, 2012).

#### Fitting of the RWLM Model

In order to determine values of parameters α and β in the RWLM model we made use of a grid search approach that maximized the probability of the model to capture the observed data. Thus, initial interval values for the grid were selected so that maximum probability was attained. Analysis of behavioral data as well as calculation of RWLM parameters was performed by using MATLAB scripts (MathWorks, Natick, MA, United States).

### Statistical Analysis

We employed non-parametric tests as our data showed significant deviation from normality as indicated by the Shapiro–Wilk test. In particular, Mann–Whitney *U*-test was utilized for comparisons between unpaired groups (CTRL vs. MED-OFF, CTRL vs. MED-ON), while comparison between paired groups (MED-OFF vs. MED-ON) was performed by the Wilcoxon signed-rank test.

Effect of dopaminergic medication on different RL parameters was addressed by the Friedman test. In order to determine a relationship between categorical variables (confidence rating and accuracy), we made use of the Chi-square test. Comparison between conditions of the RWLM parameters was achieved by the Mann–Whitney *U*-test.

Linear regression models with dependent and independent variables (meta-d and d) were also calculated.

Comparison of correlation coefficients between conditions was performed by means of the cocor package (Diedenhofen and Musch, 2015).

Statistical analyses were performed by using the software IBM SPSS Statistics (Version 24, IBM Software, Business and analytics, Armonk, NY, United States). The significance level for all two-tailed statistical tests was set up at 0.05. In order to cope with the issue of multiple comparisons, we applied the Holm correction (By considering *m* = 30) *p*-values ordered from smallest to largest, *p*^∗^ is the smallest *p*-value that satisfies the condition $p_{k}\;>\;\frac{\alpha }{m+1-k}$, where *k* is the *p*-value index), thus any *p*-value <*p*^∗^ = 0.041 was considered significant.

## Results

### Reversal Learning Parameters

**FIGURE 2:**
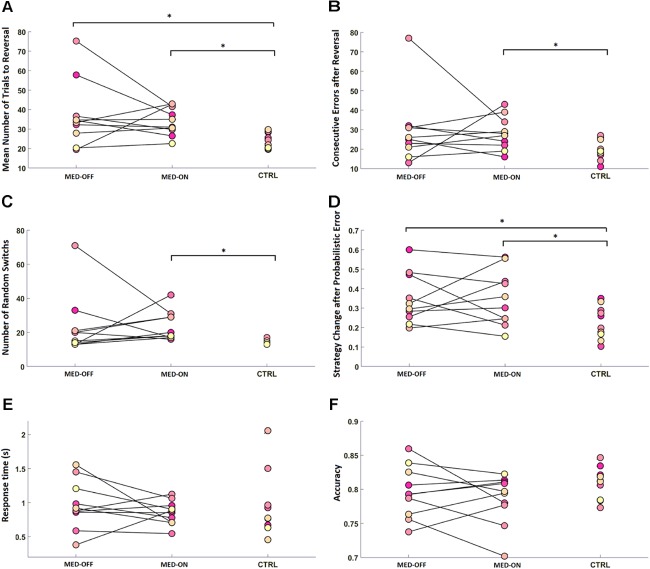
Reversal learning (RL) results for each subject across conditions [MED-OFF, MED-ON, and Controls (CTRL)]: **(A)** mean number of trials to reach reversal, **(B)** number of consecutive errors after reversal, **(C)** number of random switches, reflecting a change in decision regardless of positive feedback and prior realization of a reversal pattern, **(D)** strategy change after probabilistic error, reflecting changes in decision due to negative feedback regardless of prior realization of a reversal pattern, **(E)** response time, **(F)** accuracy. ^∗^ means statistically significant difference.

Analysis of RL data showed a significant effect of disease on RL parameters. In particular, the Mann–Whitney *U*-test revealed a significant difference in mean number of trials to reach reversal between CTRL (23.84 ± 4.03) and MED-ON (33.98 ± 7.09) (*U* = 8.50, *p* = 0.002) as well as between CTRL (23.84 ± 4.03) and MED-OFF (37.18 ± 17.03) (*U* = 20, *p* = 0.023) (**Figure 2A**). Likewise, a significant difference in number of consecutive errors after reversal (*U* = 20, *p* = 0.023) between CTRL (19.7 ± 5.17) and MED-ON (28.1 ± 8.57) was revealed (**Figure 2B**). In the same token, we found a significant difference in random switch between CTRL (15 ± 1.57) and MED-ON (23.7 ± 8.64) (*U* = 6, *p* = 0.001) (**Figure 2C**). Note that the Holm correction for multiple comparisons was applied. **Table 3** summarizes all statistical results for RL and performance.

**Table 3:** Summary of statistical results for the comparison between conditions MED-OFF, MED-ON, CTRL for RL and performance parameters.

Parameters	MED-ON vs. MED-OFF	MED-ON vs. CTRL	MED-OFF vs. CTRL
Mean number of trials to reversal	*Z* = -0.357	*U* = 8.50	*U* = 20.0
	*p* = 0.721	*p* = 0.002	*p* = 0.023
Consecutive errors after reversal	*Z* = -0.102	*U* = 20	*U* = 27.0
	*p* = 0.919	*p* = 0.023	*p* = 0.082
Number of random switches	*Z* = -0.664	*U* = 6.0	*U* = 38.5
	*p* = 0.507	*p* = 0.001	*p* = 0.378
Strategy change after probab. error	*Z* = -0.51	*U* = 25.0	*U* = 23.0
	*p* = 0.959	*p* = 0.059	*p* = 0.041
Response time	*Z* = -0.968	*U* = 49.0	*U* = 46.0
	*p* = 0.333	*p* = 0.94	*p* = 0.762
Accuracy	*Z* = -0.764	*U* = 30.0	*U* = 39.0
	*p* = 0.445	*p* = 0.131	*p* = 0.406
Mean confidence level	*Z* = -1.172	*U* = 31.5	*U* = 35.5
	*p* = 0.241	*p* = 0.162	*p* = 0.273


*Note that p-values <p^∗^ = 0.041 according to the Holm correction for multiple comparisons are considered significant. Mann–Whitney U-test was utilized for comparisons between unpaired groups (CTRL vs. MED-OFF, CTRL vs. MED-ON). The Wilcoxon signed-rank test was performed for comparison between paired groups (MED-OFF vs. MED-ON).*

### Effect of Medication on RL Parameters

No significant effect of medication was revealed in any of the RL parameters as indicated by the Friedman test.

### Performance

**FIGURE 3:**
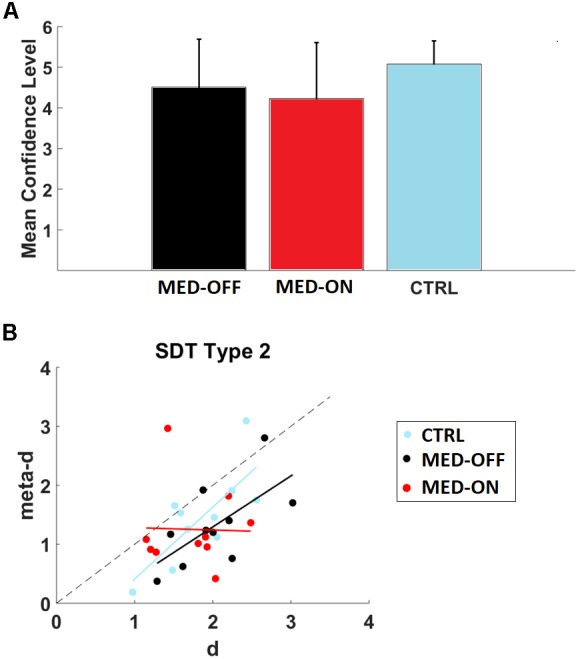
**(A)** Mean confidence level for conditions (MED-OFF, MED-ON, and CTRL); **(B)** Assessment of relative type 2 sensitivity: meta-d (observed type 2 sensitivity) and d (type 1 sensitivity) for each subject across conditions (MED-ON, MED-OFF, CTRL). Computation of such parameters was performed by using the code provided by Maniscalco and Lau (2012). Note that if meta-d = d, then a subject is metacognitively “ideal,” while the degree to which meta-d is smaller than d reflects the degree to which the subject is metacognitively inefficient. Linear regression models with dependent and independent variables (meta-d and d) for conditions: MED-ON [meta-d = -0.0401^∗^d+1.3215, *r*^2^=0.00068, *p* = 0.943 (slope)], MED-OFF [meta-d = 0.8660^∗^d-0.4393, *r*^2^=0.4292, *p* = 0.040 (slope)], and CTRL [meta-d = 1.2186^∗^d-0.8087, *r*^2^=0.5795, *p* = 0.011(slope)] are also depicted.

We found no significant difference in RT and accuracy between MED-OFF, MED-ON and CTRL (**Figures 2E,F** and **Table 3**). Nevertheless, **Figure 2F** reveals that the CTRL group presented less variability in accuracy in comparison to MED-OFF and MED-ON. Interestingly, the mean RT was more variable in controls that in MED-OFF and MED-ON groups (**Figure 2E**). We also found no significant difference in mean confidence level between conditions (**Figure 3A** and **Table 3**).

### Metacognition

We determined metacognition type 2 sensitivity (meta-d) and stimulus discrimination sensitivity (d) for each participant by using type 2 SDT. In particular, we found a significant positive correlation between d and meta-d in the case of MED-OFF (*r* = 0.655, *p* = 0.040) and CTRL (*r* = 0.761, *p* = 0.011). In line with this, a significant association between accuracy and decision confidence level was revealed by the chi-square test for MED-OFF (*df* = 5, *p* = 0.002) and CTRL (*df* = 5, *p* < 0.001). No significant correlation between d and meta-d was found for MED-ON (*r* = -0.026, *p* = 0.943) as well as not significant association as revealed by the chi-square (*df* = 5, *p* = 0.090). By using the cocor package (Diedenhofen and Musch, 2015), we compared the mentioned correlations. We found a significant difference in correlation coefficients between MED-OFF and MED-ON (*z* = 2.0749, *p* = 0.038). We found no significant difference in correlation coefficients between MED-ON and CTRL (*z* = -1.9168, *p* = 0.0553) as well as MED-OFF and CTRL (*z* = -0.4014, *p* = 0.6881).

**Figure 3B** and its legend depict the corresponding linear regression models for the mentioned correlations.

As indicated in **Figure 3B**, three subjects in CTRL outperformed or were closely in agreement with SDT expectation (meta-d ≥ d), while this occurred for two patients in MED-ON and MED-OFF, respectively. The Wilcoxon signed-rank test with the Holm correction revealed that the mean level of meta-d (1.318957) was significantly lower than the mean d (2.030269), *Z* = -2.497, *p* = 0.013 for MED-OFF. Neither significant difference between the mean level of meta-d (1.451601) and mean d (1.854869) for CTRL (*Z* = -1.988, *p* = 0.047), nor significant difference between the mean level of meta-d (1.25169) and mean d (1.742556) for MED-ON (*Z* = -1.886, *p* = 0.059) was found.

The mean values of meta-d/d were 0.64964 (MED-OFF), 0.718307 (MED-ON), and 0.782589 (CTRL) indicating that for MED-OFF on average patients exhibited absolute type 2 sensitivity at 64.96% of what would have been expected from their type 1 task performance, while such average increased to 71.83 and 78.25% for MED-ON and CTRL, respectively.

### Learning Model

**FIGURE 4:**
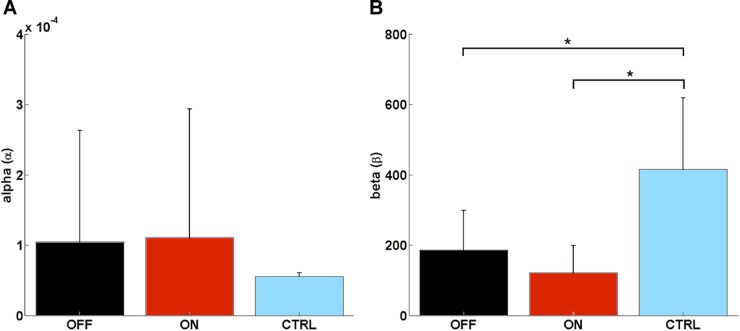
Computed parameters of the Rescorla–Wagner Model: **(A)** mean rate of learning (α), **(B)** mean decision parameter (β). Such parameters were calculated by using a grid search approach when considering initial interval values [0.600] and [0, 0.0001] for β and α, respectively. ^∗^ means statistically significant difference.

We found no significant difference in learning rate (α) between MED-ON and MED-OFF (*Z* = -0.306, *p* = 0.76), MED-ON and CTRL (*U* = 30, *p* = 0.131), MED-OFF and CTRL (*U* = 40.5, *p* = 0.473) (**Figure 4A**). However, we found a significant difference in the decision parameter (β) between MED-ON and CTRL (*U* = 6, *p* < 0.0001) and between MED-OFF and CTRL (*U* = 11, *p* = 0.003) (**Figure 4B**). The mean values of the decision parameter suggest deterministic decision making for CTRL (β = 416.33 ± 213.88) and more exploratory decision making for PD patients (MED-OFF: β = 186.05 ± 118.82; MED-ON: β = 121.28 ± 81.87).

## Discussion

The present study addressed for the first time the effect of levodopa and PD on metacognition within the framework of a RL paradigm.

In agreement with previous reports that stressed impairment of behavioral adaptation in PD patients (Cools et al., 2002; Peterson et al., 2009), we also found an effect of PD as reflected in RL behavioral parameters.

Previous studies reported on differential effects of l-dopa on the performance of a reward-based learning task with dynamic (detrimental effect) and constant (beneficial effect) reward contingencies (Graef et al., 2010), however, we found no significant effect of antiparkinsonian medication in any of the addressed parameters. Here, it is worth emphasizing that MED-ON state for PD patients in the present study was not only triggered by administration of l-dopa (7/10 patients), but also by a combination of l-dopa and dopamine agonists (3/10 patients). By grouping PD patients according to medication type (with or without agonist), we found no significant difference in RL, performance and modeling parameters. Thus, it is tempting to speculate that the presence of agonists to trigger MED-ON state plays no role on behavioral adaptation in PD as reflected by the RL parameters. Nevertheless, due to our small and unbalanced sample size, such statement must be taken with caution. Future PD studies should consider the separate effect of l-dopa and dopamine agonists in RL as also emphasized by previous reports (Moustafa, 2011).

A significant association between accuracy and confidence level was revealed for MED-OFF and CTRL but not for MED-ON. In this respect, it is tempting to speculate about involvement of a more exploratory or impulsive decision making observed in PD patients under effect of dopaminergic medication (Weiss and Marsh, 2012). However, MED-ON patients were in average less confident (**Figure 3A**), contrary to what is commonly observed that PD patients under dopaminergic medication are more confident, and less accurate (**Figure 2F**) in comparison to MED-OFF and CTRL. Noteworthy, MED-ON patients also displayed higher variability in both parameters, which may have contributed to the lack of association.

According to the Wilcoxon test with “Holm correction,” mean (meta-d) was significantly lower than mean (d) for MED-OFF, which did not hold for MED-ON and CTRL. A possible reason for this finding is that PD patients in MED-OFF displayed higher variability in accuracy than MED-ON and CTRL, although they displayed higher confidence than MED-ON but less confidence than CTRL. Such observation is in line with previous studies emphasizing impaired RL in PD (Cools et al., 2002; Peterson et al., 2009).

It is worthy to note that few subjects showed meta-d ≥ d for each condition. This is in line with the observation by Maniscalco and Lau (2012) that it is typically expected that meta-d ≤ d under the assumption that information available for the type 1 task is exhaustive of the information available for the type 2 task.

The calculated RWLM parameters indicated no significant difference in learning rate (α) between PD patients and healthy controls, although a significant exploratory behavior was highlighted for PD patients with respect to healthy controls as indicated by a significant difference in the decision parameter (β) (**Figure 4**). This was supported by parameters that describe impulsive behavior, such as random switch and percentage of strategy change, which were generally higher in PD patients (MED-ON and MED-OFF) in comparison to CTRL (**Figures 2C,D**).

Among the limitations of the present study are the small sample size and the fact MED-ON state was not triggered with the same dopaminergic medications in all the patients. To overcome these issues and therefore gain better understanding about heterogeneity across PD patients, future multicenter studies will be required.

Taken together, the present findings support previous reports on RL deficits in PD patients. Our analysis of metacognitive sensitivity (type 2 SDT) revealed significant underperformance of MED-OFF patients. Such underperformance was not significant for the conditions MED-ON and CTRL, thus suggesting that dopaminergic medication provided a non-compromising positive effect on metacognition for PD patients. Interestingly, previous studies have indicated prospective effects of dopaminergic medication on improvement of self-awareness and metacognition in healthy subjects, as reflected in a paralimbic network, e.g., medial prefrontal and medial parietal/posterior cingulate cortices (Joensson et al., 2015). In relation to this, it has been shown that dopaminergic medication influences the resilience of new information (Vilares and Kording, 2017) as indexed by improvement of performance during trials that involved decision making based on current information rather than prior information. In our RL task, dopaminergic medication did not have an effect in performance but rather in metacognitive judgment, which relied on previous information or accumulation of information that influenced each metacognitive trial. Thus, it is suggested that dopaminergic medication facilitated integration of information in the metacognitive process. Furthermore, a link between dopamine levels and the positive expectation of the outcome of one’s own actions has been shown in PD patients (Wolpe et al., 2015), namely patients on higher levodopa doses more accurately perceive the outcome of their own actions, in a way that healthy people perceive the actions of others but not themselves. In our study, a prospective improvement of the ability of self-assessment by levodopa is relevant for metacognition, e.g., subjects are more aware of their decisions via integration of information with implications in high level processing.

From a rehabilitation perspective, our results emphasize the importance of dopaminergic medication in boosting metacognitive self-awareness in PD. The variability on the values of RL and performance parameters that we obtained in the present study highlights the importance of considering neurophysiological, neuropsychiatric, functional, and anatomical factors to get deeper understanding of the effect of levodopa and PD on metacognition.

## Author Contributions

All authors contributed to the conception and design of the work. CT, MB, and JR contributed to the acquisition of data. CT, MB, KN’D, and LW contributed to analysis of data. All authors contributed to the interpretation of data and drafting, revising and approving the manuscript for publication. All authors are in agreement to be accountable for all aspects of the work.

## Conflict of Interest Statement

The authors declare that the research was conducted in the absence of any commercial or financial relationships that could be construed as a potential conflict of interest.

## Footnotes

1 http://psychtoolbox.org/

2 https://hannekedenouden.ruhosting.nl/RLtutorial/html/RL_topPage.html
